# Supplementary material for: Regulation of Alcohol and Acetaldehyde Metabolism by a Mixture of Lactobacillus and Bifidobacterium Species in Human
Source: Nutrients. 2021 May 30;13(6):1875. doi: 10.3390/nu13061875 (PMC8228388; doi:10.3390/nu13061875)
Supplement: Supplementary file 1 [file nutrients-13-01875-s001.zip › nutrients-1228905-supplementary.pdf]

Supplementary materials

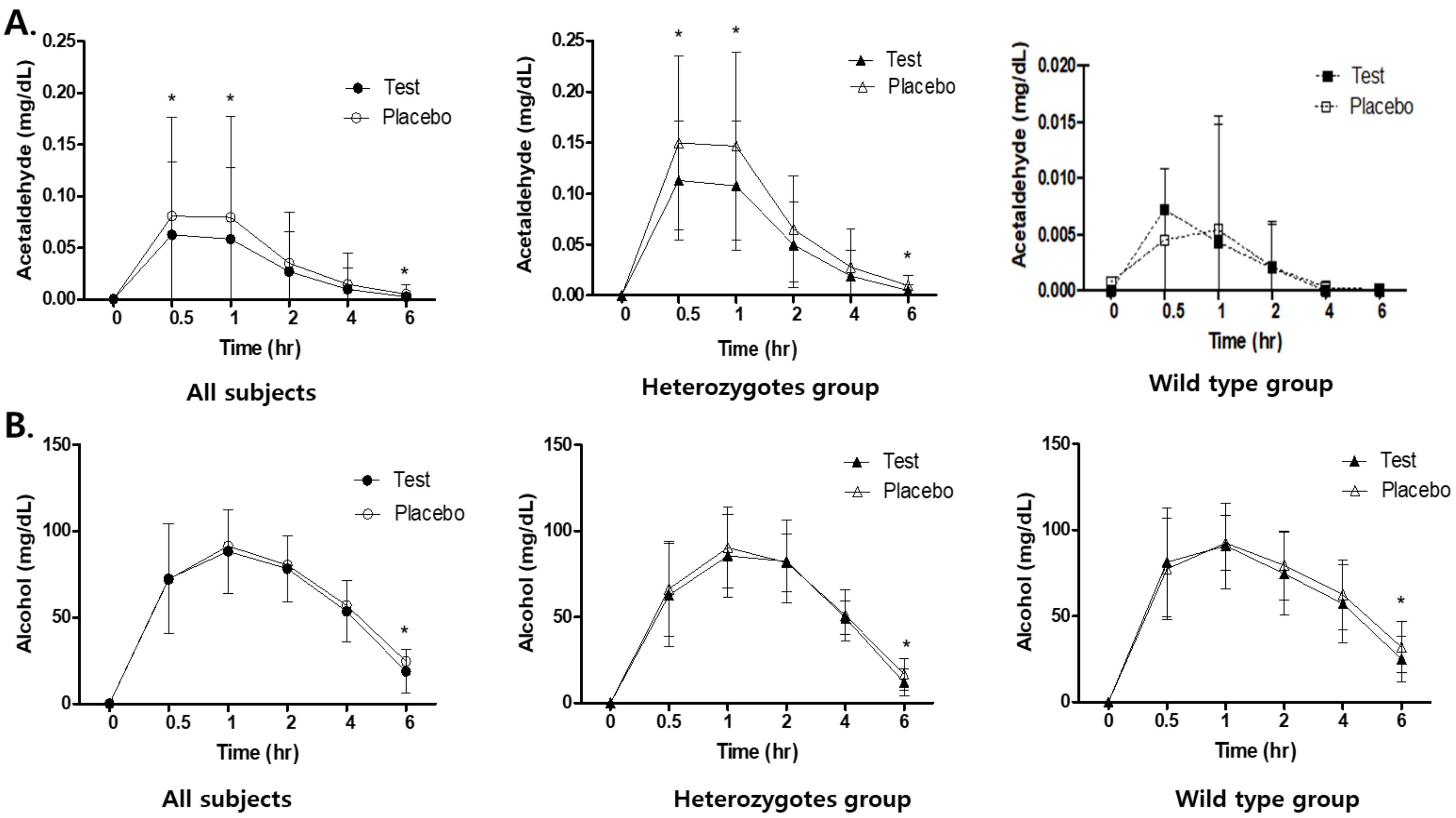

Figure S1. Changes in blood acetaldehyde (A) and alcohol (B) concentrations flowing alcohol challenge test after 15 days of supplementation.*: $p<0.05$

Table S1. Score of alcohol hangover questionnaire after 15 days of supplementation.

	Wild type (*ALDH2*1/*1*)			Heterozygote (*ALDH2*2/*1*)			Total group		
**Hangover symptom index**	**Duolac ProAP4 group (n=19)**	**Placebo group (n=19)**	***p*-value[1)]**	**Duolac ProAP4 group (n=21)**	**Placebo group (n=21)**	***p*-value[1)]**	**Duolac ProAP4 group (n=40)**	**Placebo group (n=40)**	***p*-value[1)]**
Excessive thirst	2.05±1.03	2.16±1.07	0.578	2.67±1.11	2.76±0.83	0.680	2.38±1.10	2.48±0.99	0.500
Sleepiness	2.84±1.12	2.89±1.29	0.841	3.62±1.16	3.71±0.85	0.693	3.25±1.19	3.33±1.14	0.667
Headache	2.63±1.34	2.68±1.29	0.790	2.76±0.94	2.86±1.15	0.715	2.70±1.14	2.78±1.21	0.645
Dizziness	1.84±1.07	2.42±1.07	0.053	2.57±1.03	2.52±1.12	0.853	2.23±1.10	2.48±1.09	0.201
Vomiting, a sense of helplessness	1.37±0.83	1.47±0.70	0.630	1.81±0.98	1.62±0.80	0.258	1.60±0.93	1.55±0.75	0.711
Lack of energy	1.68±0.58	1.89±0.88	0.360	2.14±1.20	2.38±1.07	0.234	1.93±0.97	2.15±1.00	0.130
Abdominal pain	1.05±0.23	1.05±0.23	>.999	1.19±0.40	1.29±0.56	0.493	1.13±0.33	1.18±0.45	0.534
Diarrhea	1.05±0.23	1.32±0.67	0.135	1.05±0.22	1.14±0.48	0.428	1.05±0.22	1.23±0.58	0.090
Concentrati on difficulty	1.74±0.87	1.95±0.71	0.331	2.00±1.22	2.24±1.14	0.366	1.88±1.07	2.10±0.96	0.183
More sensitive to irritation than usual (light and sound)	1.26±0.45	1.32±0.48	0.667	1.33±0.66	1.62±0.92	0.162	1.30±0.56	1.48±0.75	0.147
Sleep difficulty	1.32±0.82	1.16±0.50	0.380	1.05±0.22	1.24±0.54	0.104	1.18±0.59	1.20±0.52	0.812
Sweat more than usual (sticky sweat)	1.42±0.61	1.16±0.37	0.056	1.48±0.98	1.33±0.66	0.576	1.45±0.81	1.25±0.54	0.173

Melancholy	1.16±0.69	1.00±0.00	0.331	1.05±0.22	1.10±0.30	0.576	1.10±0.50	1.05±0.22	0.570
Memory disconnection	1.16±0.37	1.53±1.12	0.185	1.05±0.22	1.14±0.36	0.329	1.10±0.30	1.33±0.83	0.107
Heart palpitations	1.32±0.58	1.11±0.32	0.163	3.24±1.04	2.62±1.36	0.024	2.33±1.29	1.90±1.26	0.008
Zone (nausea = feels like vomiting is urgent)	1.26±0.56	1.32±0.58	0.749	1.57±0.81	1.57±0.98	>.999	1.43±0.71	1.45±0.81	0.838
Elated mood	2.00±1.00	1.79±0.92	0.494	2.57±1.21	2.05±1.12	0.018	2.30±1.14	1.93±1.02	0.042
Blush of the face	1.63±0.90	1.58±0.84	0.772	4.00±1.14	3.81±1.08	0.446	2.88±1.57	2.75±1.48	0.418
Body warms up	1.79±0.85	1.63±0.83	0.604	3.52±1.29	3.29±1.15	0.309	2.70±1.40	2.50±1.30	0.282
Shortness of breath	1.11±0.32	1.00±0.00	0.163	2.57±1.29	2.33±1.06	0.204	1.88±1.20	1.70±1.02	0.090
Symptom index (20)*	31.68±8.09	32.42±6.99	0.752	43.24±10.14	42.62±10.46	0.734	37.75±10.82	37.78±10.26	0.986
Symptom index (13)#	21.26±5.09	22.84±5.25	0.305	24.71±6.01	25.71±6.22	0.395	23.08±5.79	24.35±5.89	0.174
Symptom index (7)†	12.21±3.14	12.89±3.49	0.407	14.76±4.35	15.52±3.83	0.339	13.55±3.99	14.28±3.86	0.197

Values are presented as mean ± SD. 1) Analyzed using paired *t*-test (compared between groups) * Excessive thirst, sleepiness, headache, dizziness, vomiting, a sense of helplessness, lack of energy, abdominal pain, diarrhea, concentration difficulty, more sensitive to irritation than usual (light and sound), sleep difficulty, sweat more than usual (sticky sweat), melancholy, memory disconnection, heart palpitations Zone (nausea = feels like vomiting is urgent), elated mood, blush of the face, body warms up, shortness of breath. # Excessive thirst, sleepiness, headache, dizziness, vomiting, a sense of helplessness, abdominal pain, diarrhea, concentration difficulty, more sensitive to irritation than usual (light and sound), sweat more than usual (sticky sweat), melancholy, memory disconnection.

† Excessive thirst, sleepiness, headache, dizziness, a sense of helplessness, concentration difficulty, heart palpitations, zone (nausea = feels like vomiting is urgent).
